# Verbascoside inhibits progression of glioblastoma cells by promoting Let‐7g‐5p and down‐regulating HMGA2 via Wnt/beta‐catenin signalling blockade

**DOI:** 10.1111/jcmm.14884

**Published:** 2020-01-30

**Authors:** Wei‐Qiang Jia, Jian‐Wei Zhu, Cheng‐Yong Yang, Jun Ma, Tian‐You Pu, Guo‐Qiang Han, Ming‐Ming Zou, Ru‐Xiang Xu

**Affiliations:** ^1^ Department of Neurosurgery Sichuan Provincial People's Hospital University of Electronic Science and Technology of China Chengdu China; ^2^ Department of Neurosurgery Guizhou Provincial People's Hospital Guiyang China; ^3^ Department of Neurosurgery The Seventh Medical Center of PLA General Hospital Beijing China

**Keywords:** autophagy, glioblastoma, invasion, let‐7g‐5p, migration, verbascoside, viability, Wnt/β‐catenin signalling pathway

## Abstract

Glioblastoma (GBM) continues to show a poor prognosis despite advances in diagnostic and therapeutic approaches. The discovery of reliable prognostic indicators may significantly improve treatment outcome of GBM. In this study, we aimed to explore the function of verbascoside (VB) in GBM and its effects on GBM cell biological processes *via* let‐7g‐5p and HMGA2. Differentially expressed GBM‐related microRNAs (miRNAs) were initially screened. Different concentrations of VB were applied to U87 and U251 GBM cells, and 50 µmol/L of VB was selected for subsequent experiments. Cells were transfected with let‐7g‐5p inhibitor or mimic, and overexpression of HMGA2 or siRNA against HMGA2 was induced, followed by treatment with VB. The regulatory relationships between VB, let‐7g‐5p, HMGA2 and Wnt/β‐catenin signalling pathway were determined. The results showed that HMGA2 was a direct target gene of let‐7g‐5p. VB treatment or let‐7g‐5p overexpression inhibited HMGA2 expression and the activation of Wnt/β‐catenin signalling pathway, which further inhibited cell viability, invasion, migration, tumour growth and promoted GBM cell apoptosis and autophagy. On the contrary, HMGA2 overexpression promoted cell viability, invasion, migration, tumour growth while inhibiting GBM cell apoptosis and autophagy. We demonstrated that VB inhibits cell viability and promotes cell autophagy in GBM cells by up‐regulating let‐7g‐5p and down‐regulating HMGA2 via Wnt/β‐catenin signalling blockade.

## 1 INTRODUCTION

As one of the most commonly occurring malignancies, glioblastoma (GBM) continues to impose significant healthcare burden worldwide.^1^ Currently, GBM treatment options typically include maximally safe surgical resection with adjuvant radiotherapy.^2^ Owing to the high usual age of onset, tumour location and poor understanding of GBM pathophysiology, mean survival is approximately only 16 months despite optimal treatment.^3^ Regrettably, advances in therapeutic strategies have not translated to increase in the survival rate of patients with GBM.^4^ As such, the development of new diagnostic and therapeutic approaches demands improved understanding of the molecular mechanisms that underpin this condition, including both genetic and epigenetic factors.^5^

Verbascoside (VB) is a type of herb that has recently received research attention owing to its significant clinical value.^6^ Known as an antioxidant, VB has been widely investigated owing to its purported biological and pharmacological capabilities, which include neuroprotective, hepatoprotective, antineoplastic and antiproliferative effects.^7^ VB has been shown to alleviate muscle fatigue and reduce oxidative stress brought on by exhaustive exercise through its effect on free radicals and lipid peroxidation.^8^ Additionally, VB has exhibited anti‐tumour activities. It has been found to significantly decrease tumour cell viability and growth and enhance tumour cell apoptosis in leukaemia.^9^ As a member of microRNAs, let‐7g‐5p is poorly expressed in GBM and suppressed during GBM cell invasion and migration by binding to VSIG4 and blocking the epithelial‐mesenchymal pathway.^10^ High mobility group A2 (HMGA2), a high mobility group AT‐hook (HMGA) protein, has been found to be overexpressed during tumour progression while influencing several gene expression patterns that impact biological processes such as cell survival, differentiation and apoptosis.^11^ HMGA2 serves as a competitive endogenous RNA and was found to play a role in lung cancer progression and metastasis, acting via its regulatory network.^12^ Reports have also indicated that HMGA2 induces cell invasion and metastasis via the TGFβ signalling pathway, which has been demonstrated in colon and breast cancer.^13^ Previous studies have also revealed that several miRNAs could target HMGA2, thus exerting its regulatory functions in many different types of cancers.^14^, ^15^ In GBM, the Wnt/β‐catenin signalling pathway is known to act as a vital downstream target.^16^ Reports have indicated that Sohlh1 participates in the progression of GBM through its effect on the Wnt/β‐catenin signalling pathway.^17^ Therefore, our study, in order to identify a novel molecular target for GBM management, framed a hypothesis that VB could repress GBM cell invasion, migration and tumour growth by inhibiting let‐7g‐5p‐mediated HMGA2 *via* the Wnt/β‐catenin signalling pathway.

## 2 MATERIALS AND METHODS

### 2.1 Ethics statement

All animal experimental procedures were conducted after the approval of the Animal Committee of Sichuan Provincial People's Hospital, University of Electronic Science and Technology of China and the Seventh Medical Center of PLA General Hospital.

### 2.2 In silico analysis

miRNA expression microarray data of GBM were obtained from the Gene Expression Omnibus (GEO) database (https://www.ncbi.nlm.nih.gov/geo/). Differences in miRNA expression between normal samples and tumour samples in the microarray data were determined using the GEO2R tool, and the log fold change value of differentially expressed miRNAs was analysed.

### 2.3 Cell culture

Glioblastoma cell lines A172, SHG139, SHG‐44, U87 and U251 were purchased from Shanghai Institutes for Biological Sciences, Chinese Academy of Sciences, (Shanghai, China). The cells were grown in Dulbecco's Modified Eagle's Medium (DMEM; Sigma) containing 10% foetal bovine serum (FBS), 100 mg/mL streptomycin and penicillin, and incubated with 5% CO_2_ in saturated humidity conditions at 37°C. Cells in the logarithmic growth phase were treated with trypsin, followed by centrifugation. After removal of the supernatant, the cells were re‐suspended, and 100 µL of suspension (5.0 × 10^4^ cells/mL) was seeded into a 96‐well plate. Twenty‐four hours after incubation, 0, 1, 20, 40, 60, 80 and 100 µmol/L VB were added into the cell suspension, in individual experiments. A blank group (cells containing DMEM only) and a negative control (NC) group (cells containing NC of the same concentration) were designed for the subsequent experiments. Each experiment was repeated three times.

### 2.4 Cell counting Kit‐8 (CCK‐8) assay

A CCK‐8 kit (Dojindo) was used to determine cell viability. GBM cell lines (A172, SHG139, SHG‐44, U87 and U251) were treated with VB at different concentrations. At approximately 80% confluence, cells were inoculated into a 96‐well plate at a plating density of 5000 cells/mL with 100 µL per well. After incubation for 24 hours, 10 μL of CCK (AbMole‐M4839, Abmole Bioscience Inc) was added to the cells in each well, followed by incubation for 1‐4 hours at 4°C. Next, 150 μL of dimethyl sulfoxide (DMSO) was added to each well followed by shaking for 10 minutes. An optical density (OD) value at 570 nm was obtained to reflect cell survival using a multimode microplate reader (SpectraMax i3x, Molecular Devices). Cell survival rate was computed as: 100% ‐ (OD value of the experimental group ‐ OD value of the blank group)/(OD value of the NC group ‐ OD value of the blank group) × 100%. IC_50_ of VB was calculated in accordance with the inhibition rate of gradient concentration. Th cell lines and drug concentrations presenting the highest IC_50_ were selected based on this screening experiment and used in further assays.

### 2.5 Dual‐luciferase reporter gene assay

According to sequences of the binding sites between 3′‐untranslated region (UTR) of HMGA2 mRNA and let‐7g‐5p, target and mutant sequences were synthesized, and Xho I and Not I endonuclease sites were created at the downstream of both sequences. The cloned product was transferred into a PUC57 carrier, followed by the application of DNA sequencing in order to detect the recombinant plasmid after it had been confirmed as a positive clone. The plasmid was amplified, and the psiCHECK‐2 vector was used (VECT90299, Huayueyang Biotechnology, Co., Ltd.) with cloning sequences inserted to escherichia coli DH5α cells. The plasmids were extracted in accordance with the instructions of the Omega Plasmid Miniprep Kit (D1100‐50T, Solarbio Life Science). Next, 293T/17 cells were seeded in a 6‐well plate at a density of 2 × 10^5^ cells/well. After cell adherence to the wells, the cells were transfected using the aforementioned methods in a reaction system containing 2 mL of the culture medium, 250 µL of Opti‐MEN and 4 µg of plasmids, followed by a 48‐h incubation. The effects of let‐7g‐5p on the luciferase activity of HMGA2 3′‐UTR were detected using a dual‐luciferase reporter gene assay kit (D0010, Solarbio Life Science) based on the manufacturer's protocol. The fluorescence intensity was assessed using a Glomax20/20 luminometer fluorescence detector (E5311, Zhongmei Biotechnology Co., Ltd., Xi'an).

### 2.6 RNA pull‐down assay

U87 cell lines were transfected with 50 nmol/L biotinylated let‐7g‐5p wild‐type (wt) and 50 nmol/L biotinylated let‐7g‐5p mutant (mut). After transfection for 48 hours, the cells were washed with phosphate‐buffered saline (PBS) and then cultured with specific lysis buffer (Ambion). Ten minutes later, 50 mL of the lysate was extracted. The remaining lysate was incubated with M‐280 streptavidin‐coated magnetic beads (Sigma‐Aldrich) containing RNase‐free and yeast transfer RNA (tRNA; Sigma‐Aldrich). After a 3‐hour incubation at 4°C, the mixture was washed twice with cold lysis buffer, thrice with low salt buffer and once with high‐salt buffer. Antagonistic let‐7g‐5p probe was regarded as the NC. Total RNA was extracted using the TRIzol method and the mRNA expression of HMGA2 was measured using reverse transcription quantitative polymerase chain reaction (RT‐qPCR).

### 2.7 Cell transfection and grouping

According to the sequences of HMGA2 obtained from the National Center for Biotechnology Information (NCBI), blank plasmids, HMGA2 vector plasmids and HMGA2 shRNA plasmids were each constructed (Sangon Biotech Shanghai Co., Ltd.).

U87 and U251 cells were divided into the following groups: blank group (cells cultured with medium), NC group (cells cultured with medium containing NC of the same concentration), VB group (cells cultured with medium containing 50 µmol/L VB), VB‐3‐methyladenine (3‐MA) group (cells cultured with medium containing 50 µmol/L VB and autophagy inhibitor 3‐MA), let‐7g‐5p mimic group (cells transfected with let‐7g‐5p mimic), let‐7g‐5p inhibitor group (cells transfected with let‐7g‐5p inhibitor), VB + let‐7g‐5p inhibitor group (cells transfected with let‐7g‐5p inhibitor in the presence of 50 µmol/L VB), NC mimic group (cells transfected with NC mimic), NC inhibitor group (cells transfected with NC inhibitor), HMGA2 vector group (cells transfected with HMGA2 vector), si‐HMGA2 group (cells transfected with HMGA2 siRNA), let‐7g‐5p mimic + HMGA2 group (cells cotransfected with let‐7g‐5p mimic and HMGA2 vector) and the let‐7g‐5p inhibitor + si‐HMGA2 group (cells cotransfected with let‐7g‐5p inhibitor and HMGA2 siRNA). U87 and U251 cells at the logarithmic growth phase were transiently transfected using Lipofectamine 2000.

### 2.8 Xenograft tumour in nude mice

The U87 and U251 cells were re‐suspended and adjusted to 1 × 10^7^ cells/mL with serum‐free Roswell royal park memorial institute (RPMI) 1640 medium (Gibco, Invitrogen). A total of 60 specific pathogen free (SPF) male nude mice aged 4 weeks, weighing 14‐16 g, (Shanghai SLAC Laboratory Animal Technology Co., Ltd.) were allowed to acclimatize for 1 week and randomly divided into 12 groups (5 nude mice per group). The left axillary part of the nude mice was injected with 1 × 10^6^ cells, and they were further injected with VB according to predefined experiment protocols. All nude mice were housed in the same environment and monitored every week. The maximum (a) and minimum diameter (b) of the tumours that appeared were measured using a vernier caliper, and the approximate volume was calculated according to the formula: V = a × b^2^/2. After injection over a 28‐day period, the nude mice were killed. The xenograft and metastatic tumours were extracted in order to determine the let‐7g‐5p expression.

### 2.9 Scratch test

After transfection for 48 hours, U87 and U251 cells were seeded in a 6‐well plate at a density of 1 × 10^5^ cells/well. When cells reached approximately 90% confluence, 4 horizontal and perpendicular thin wounds were created respectively using a sterile 200‐μL pipette applied with equal force. After discarding the original medium, the cells in each well were incubated with 2 mL of serum‐free fresh medium. The images of the cells were photographed at the 0th and 24th h under an inverted microscope. Five visual fields of each sample were selected randomly to obtain the mean value and relative widths of the scratches were also measured at multiple sites. The rate of wound healing was determined as: (width at the 0th h ‐ width at the 24th h)/width at the 0th h × 100%.

### 2.10 Transwell assay

After transfection for 48 hours, the U87 and U251 cell lines were starved in serum‐free medium for 24 hours, followed by detachment. A cell suspension (3 × 10^4^ cells/mL) was prepared using serum‐free Opti‐MEMI (31985008, Nanjing SenBeiJia Biotechnology Co., Ltd.) containing 10 g/L bovine serum albumin (BSA; 9998S, Shanghai Beinuo Biotechnology Co., Ltd.). Transwell chambers (3422, Beijing Unique Biotechnology Co., Ltd.) were placed into a 24‐well plate. The apical chambers were coated with diluted Matrigel (1:8; 40111ES08, Yeasen) and dried at room temperature. After being detached and washed twice with PBS, the cells were re‐suspended with RPMI 1640 medium (R7755, Beijing GENIA Bioscience Technology Ltd.) with cell density adjusted to 1 × 10^5^ cells/mL. A total of 200 μL of cell suspension was inoculated into the apical chambers coated with Matrigel (356234, Shanran Biotechnology Co., Ltd.). Next, 600 μL of RPMI 1640 medium with 20% BSA was added to the basolateral chambers. Twenty‐four hours after incubation, the cells in the apical chambers were removed using cotton buds, fixed with 4% paraformaldehyde for 15 minutes, stained with crystal violet solution (prepared with methanol) for 15 minutes and rinsed thrice with PBS. Five random visual fields were photographed (200×) using an inverted microscope (XDS‐800D, Shanghai Caikon Optical Instrument Co., Ltd.) to count the number of cells which had invaded the Matrigel. Three replicates were set up in each group.

### 2.11 Laser confocal microscopy

U87 and U251 cells were seeded onto cover slips in a 48‐well culture dish. After a 24‐hour treatment process with solutions, the cells were incubated with 5% CO_2_ at 37°C and fixed with 4% ice‐cold paraformaldehyde for 15 minutes. After being washed with PBS, the cells were treated with 0.1% Triton‐X‐100 for 15 minutes and cultured with medium‐diluted primary and secondary antibodies at 250 μL/well in an incubator with 5% CO_2_ at 37°C. Next, the cells were rinsed with PBS, followed by addition of LC3B polyclonal antibody (1:200; YM3381; ImmunoWay Biotechnology Company), avoiding exposure to light for 20 minutes. The cells were mounted using a drop of anti‐fluorescence quenching medium and then observed under a laser confocal microscopy (Keyence) for detection (the excitation wavelength, emission wavelength and intensity distribution for scanning of Cy3 were 543 nm, 560‐670 nm, 50; and resolution was 1024:60 n).

### 2.12 Flow cytometry

After transfection for 48 hours, the cells were treated with ethylenediaminetetraacetic acid (EDTA)‐free trypsin, collected in a flow tube and centrifuged with supernatant discarded. According to the instructions of the Annexin‐V‐fluorescein isothiocyanate (FITC) kit (C1063, China Ocean), Annexin‐V‐FITC/propidium iodide (PI) solution was prepared with Annexin‐V‐FITC, PI and N‐2‐Hydroxyethylpiperazine‐N'‐2‐Ethanesulfonic Acid (HEPES) buffer solutions at the ratio of 1:2: 50. A total of 1 × 10^6^ cells were re‐suspended with 100 μL of Annexin‐V‐FITC/PI staining solution. After incubation for 15 minutes at room temperature, 1 mL of HEPES buffer solution (GNM‐11344, Shanghai Jingke) was added to the cells, oscillated and mixed evenly. The cells were excited at 488 nm, FITC fluorescence was detected using a 525 nm band‐pass filter, whereas PI was detected using a 620 nm band‐pass filter to determine cell apoptosis.

### 2.13 RT‐qPCR

Total RNA from U87 and U251 cells was extracted using TRIzol (RP2401, BioTeKe Corporation). RT‐qPCR primers of let‐7g‐5p, LC3, Beclin1, Bax, Bcl‐2 and Caspase‐3 were designed using the Premier 5.0 software and then synthesized by Invitrogen Corp (Table 1). RNA was reversely transcribed into cDNA in accordance with the instructions of the PrimeScript RT kit (RR036A, Takara Biotechnology Co., Ltd.). The primers were amplified using a PCR instrument (7300, Applied Biosystems) in accordance with the instructions of the SYBR^®^ Premix Ex TaqTM II kit (RR820A, Takara Biotechnology Co., Ltd.). Next, 2 μg of total RNA was used as the template, glyceraldehyde‐3‐phosphate dehydrogenase (GAPDH) was regarded as the internal reference for HMGA2, β‐catenin, LC3, Beclin1, Bax, Bcl‐2, Caspase‐3 and protein kinase C (PKC) and U6 served as internal reference for let‐7g‐5p. The relative mRNA expression of these genes was determined using the 2^‐ΔΔCt^ method.^18^

**Table 1:** Primer sequences for RT‐qPCR

Genes	Forward sequences (5′‐3′)	Reserve primers (5′‐3′)
miR‐192	CTCGAGGACTGGGGAGCCCAGGTGAG	GGATCCGAGGCAGCAAATGCTTGGACAG
miR‐96	GCCCGCTTTGGCACTAGCACATT	GTGCAGGGTCCGAGGT
let‐7g‐5p	GAAGCAGCCAGCCCAAAGT	GGAGGAGCAGAACTGAACCC
U6	TTCAACTATGGCAGCTCGGT	AACAACAGAGGCCAGCGTTA
HMGA2	CACAAGAGTCTGCCGAAGAGG	GAAGAACAACGGGCTGGTCG
β‐Catenin	CTGAGATCCCCCTGCTTTCC	ATAGCCAGGGTTAGCTCAGTG
LC3	GCGAGTTACCTCCCGCAG	TCATGTTGACATGGTCCGGG
Beclin1	CTCCCGAGGTGAAGAGCATC	GCTGTTGGCACTTTCTGTGG
Bax	GCTGCCTTGGACTGTGTTTTT	CAGATGCCGAAGTGTGTCCC
Bcl‐2	GAACTGGGGGAGGATTGTGG	CATCCCAGCCTCCGTTATCC
Caspase‐3	GTCCTGGGACACCGGTTAT	ACGGCAGGCCTGAATAATGA
PKC	CTGGTCATCGCTAACATAG	GTTAAGAATCACTTCCCACT
GAPDH	AATGGGCAGCCGTTAGGAAA	GCGCCCAATACGACCAAATC

Notes

All genes above were all obtained from NCBI.

Abbreviations: Bax, Bcl‐2 associated X protein; Bcl‐2, B‐cell lymphoma‐2; Caspase, cysteinyl aspartate specific proteinase; GAPDH, glyceraldehyde‐3‐phosphate dehydrogenase; HMGA2, high mobility group A2; LC3, Autophagy marker Light Chain 3; let‐7g‐5p, microRNA let‐7g‐5p; NCBI, National Center for Biotechnology Information; PKC, protein kinase C; RT‐qPCR, reverse transcription quantitative polymerase chain reaction; U6, U6 small nuclear.

### 2.14 Western blot analysis

Total protein of the U87 and U251 cells was extracted using radio‐immunoprecipitation assay (RIPA) lysis buffer (R0010, Solarbio Life Science), with protein concentration measured using a bicinchoninic acid (BCA) protein assay kit (G3522‐1, GBCBIO Technologies). After polyacrylamide gel electrophoresis (PAGE), the protein was transferred onto nitrocellulose membranes using the wet transfer method and then blocked with 5% BSA for 1 hour at room temperature. The membranes were incubated with the following primary antibodies to LC3 (1:500, ab128025), Beclin1 (1:1000, ab62557), Bax (1:1000, ab53154), Bcl‐2 (1:1000, ab59348), Caspase‐3 (1:500, ab13847) and PKC (1:1000, ab19031) at 4°C overnight. All antibodies were purchased from Abcam. Next, the membranes were washed five times with PBS (5 minutes each time) and incubated with horseradish peroxidase (HRP)‐labelled secondary rabbit anti‐human antibody to IgG (1:5000, F020218, Baiaolaibo Science and Technology Ltd.) for 1 hour. Each membrane was developed using electrochemiluminescence (ECL) (ECL808‐25, Biomiga) for 1 minutes. After the solution was discarded, each membrane was covered by preservative film and exposed by X‐ray (36209ES01, Shanghai qcbio Science & Technologies, Co., Ltd.) in order to visualize the results. GAPDH (1:1000, ab8245, Abcam) was used as the internal reference and relative expression was computed as the ratio of the grey value of the target band to that of the internal reference band.

### 2.15 Statistical analysis

The SPSS 21.0 software (IBM Corp.) was employed for statistical analysis. All descriptive measurement data were summarized as mean ± SD. Comparisons between multiple groups were performed using one‐way analysis of variance and comparisons between two groups were analysed using the *t* test, while pairwise comparisons were performed using the least‐significant difference (LSD) test. *P* < .05 was statistically significant.

## 3 RESULTS

### 3.1 VB promotes let‐7g‐5p expression in nude mice with xenograft tumours

**Figure 1:**
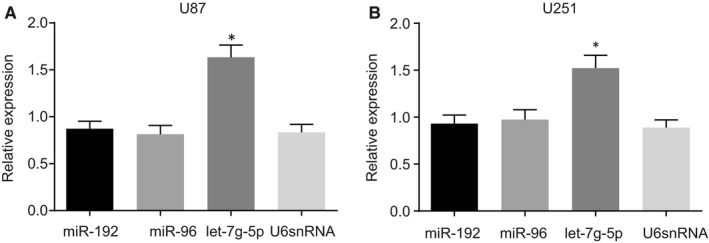
VB promotes let‐7g‐5p expression in nude mice with xenograft tumours. A, the expression of miR‐192, miR‐96, let‐7g‐5p and U6 snRNA in nude mice injected with U87 cells; B, the expression of miR‐192, miR‐96, let‐7g‐5p and U6 snRNA in nude mice injected with U251 cells; ^*^ *P* < .05 vs U6 snRNA. All measurement data were summarized as mean ± SD. Comparisons among multiple groups were performed with one‐way analysis of variance and comparisons between two groups were analysed using a *t* test. The experiment was repeated three times independently. VB, Verbascoside

A GBM‐related microarray data set ‘GSE103228’ was obtained from the GEO database. Differentially expressed miRNAs in tumour samples versus those in normal samples were determined and it was found that miR‐192, miR‐96, and let‐7g‐5p were each differentially expressed in the GBM samples (Table 2), consistent with other reports of these three miRNAs playing important roles in drug response of tumours.^19^, ^20^, ^21^, ^22^ However, as it remained unclear whether these three miRNAs were affected by VB in GBM, the expression of miR‐192, miR‐96 and let‐7g‐5p in nude mice with xenograft GBM tumours was determined using RT‐qPCR. As shown in Figure 1A,B, miR‐192, miR‐96, and let‐7g‐5p were each highly expressed in nude mice with xenograft tumours. However, let‐7g‐5p was expressed at significantly higher levels in nude mice treated with VB (*P* < .05). These results indicated that VB was a promoter of let‐7g‐5p.

**Table 2:** Expression of miRNAs in GSE103228

miRNAs	LogFC	Up‐regulation/Down‐regulation
hsa‐miR‐192‐5p	1.253385	Up‐regulation
hsa‐miR‐96‐5p	−3.161342	Down‐regulation
hsa‐let‐7g‐5p	−2.621895	Down‐regulation

Abbreviations: FC, fold change; hsa, homo sapiens; miR, miRNAs.

### 3.2 VB has stronger inhibitory ability in cell viability of U87 and U251 cells than in A172, SHG139 and SHG‐44 cells

**Figure 2:**
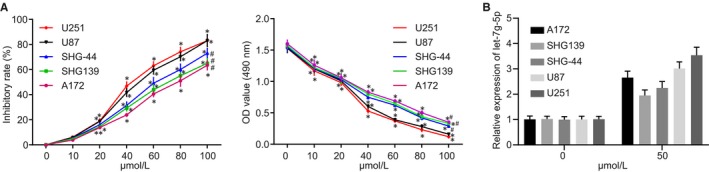
The inhibitory rate of U87 and U251 cells is higher in all concentrations of VB in comparison with A172, SHG139 and SHG‐44 cells at 72 h after VB treatment. A, Effects of different concentrations of VB (0, 10, 20, 40, 60, 80, 100 µmol/L) on the growth inhibition curve and cell proliferation of A172, SHG139, SHG‐44, U87 and U251 cells at 72 h. B, Statistics of A. ^*^ *P* < .05 vs highest VB concentration at the same time‐point; ^#^ *P* < .05 vs the U87 and U251 cells. All measurement data were summarized as mean ± SD. Repeated measures analysis of variance was used for comparison of values at different time‐points. The experiment was repeated three times independently

The cell viability of A172, SHG139, SHG‐44, U87 and U251 cells was determined after treatment with varying concentrations of VB using CCK‐8. The results indicated that viability of A172, SHG139, SHG‐44, U87 and U251 cells exhibited the same trend upon treatment with different concentrations of VB (0, 10, 20, 40, 60, 80 and 100 µmol/L) at 72 hours. The inhibitory rate was calculated based on the CCK‐8 assay where cell viability was reflected by the OD value of each well at 570 nm (Figure 2A,B). VB had an inhibitory effect on the viability of A172, SHG139, SHG‐44, U87 and U251 cells. When the concentration of VB was 0, cell viability and proliferation reached their respective maximum values. As the concentration of VB increased, cell viability and proliferation were decreased, with an elevated inhibitory rate. In comparison with A172, SHG139 and SHG‐44 cells, the recorded inhibitory rate of VB for the U87 and U251 cells was greater, suggesting higher sensitivity, and in consistence, the observed cell viability was lower (all *P* < .05). When the concentration of VB applied ranged 40‐60 µmol/L, a statistically significant effect was notable (*P* < .05). The IC_50_ values of A172, SHG139, SHG‐44, U87 and U251 cells were 87.36, 81.11, 72.34, 52.94 and 50.32 μmol/L, respectively. Therefore, U87 and U251 cell were selected for subsequent experiments, using a VB concentration of 50 µmol/L. Thereafter, let‐7g‐5p expression after VB treatment was detected by RT‐qPCR. The results showed that let‐7g‐5p expression was up‐regulated upon VB treatment. In addition, the content of let‐7g‐5p in U87 and U251 cells was higher than that in A172, SHG139 and SHG‐44 cells (Figure 2C).

### 3.3 VB suppresses tumour growth of GBM in nude mice

**Figure 3:**
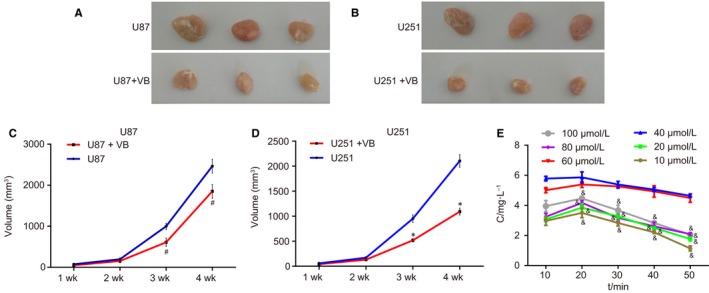
VB inhibits the growth of GBM U87 and U251 xenograft tumours in nude mice. A, xenograft tumours in the U87 and U87 + VB groups; B, xenograft tumours in the U251 and U251 + VB groups; C, changes of tumour volume at 1, 2, 3 and 4 w in the U87 and U87 + VB groups; D, changes of tumour volume at 1, 2, 3 and 4 w in the U251 and U251 + VB groups; E, blood concentration‐time curve after VB injection; ^*^ *P* < .05 vs the U87 group; ^&^ *P* < .05 vs last blood concentration at the same time; ^#^ *P* < .05 vs the U251 group. All measurement data were summarized as mean ± SD. Repeated measures analysis of variance was used for comparison of values at different time‐points. The experiment was repeated three times independently. GBM, glioblastoma

U87 and U251 xenograft GBM tumours were induced in nude mice to study the effects of VB on the in vivo tumour growth. In comparison with the U87 group, tumour growth and volume during the first two weeks did not differ remarkably in the U87 + VB group (*P* > .05), and tumour growth was suppressed after VB injection (*P* < .05; Figure 3A‐C). Similarly, as compared to the U251 group, tumour growth and volume in the first two weeks did not differ remarkably in the U251 + VB group (*P* > .05), while tumour growth was suppressed after VB injection (*P* < .05; Figure 3B‐D). After VB injection, the average blood concentration was determined using high‐performance liquid chromatography (HPLC), and the blood concentration‐time curve is illustrated in Figure 3E. No differences were observed at VB concentrations of 40 and 60 µmol/L (*P* > .05), but blood concentration differed significantly at other concentrations (all *P* < .05). Based on these data, a concentration of 50 µmol/L was designed for the subsequent experiments involving VB.

### 3.4 HMGA2 is confirmed as a target gene of let‐7g‐5p

**Figure 4:**
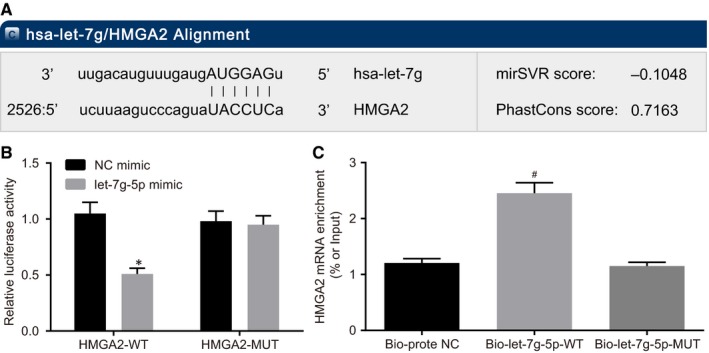
HMGA2 is the target gene of let‐7g‐5p. A, binding sites between HMGA2 and let‐7g‐5p; B, luciferase activity of the HMGA2‐Wt and HMGA2‐Mut after transfection; C, HMGA2 mRNA enrichment of bio‐let‐7g‐5p‐wt and bio‐let‐7g‐5p‐mut; ^*^ *P* < .05 vs the NC mimic group; ^#^ *P* < .05 vs the bio‐probe NC group. All measurement data were summarized as mean ± SD. Comparisons among multiple groups were performed with one‐way analysis of variance. The experiment was repeated three times independently. HMGA2, high mobility group A2; NC, negative control; wt, wild‐type; mut, mutant; 3′UTR, 3′ untranslated region

Subsequently, let‐7g‐5p was examined in order to determine whether it could directly regulate HMGA2 using in silico target prediction and luciferase activity determination. The web‐based bioinformatics prediction software indicated the presence of a target‐binding site between HMGA2 and let‐7g‐5p, confirming HMGA2 as a target of let‐7g‐5p (Figure 4A). Luciferase activity determination further demonstrated that let‐7g‐5p directly targeted HMGA2. As shown in Figure 4B, cells treated with wt‐let‐7g‐5p and HMGA2 plasmid showed significantly decreased luciferase activity when cotransfected with let‐7g‐5p mimic compared to co‐transfection with NC mimic (*P* < .05). However, the luciferase activity of the cells treated with mut‐let‐7g‐5p and HMGA2 plasmid did not differ significantly among groups (*P* > .05). These findings suggested that let‐7g‐5p could specifically bind to HMGA2.

The results of RNA pull‐down assay (Figure 4C) revealed that HMGA2 mRNA was expressed at a higher level in the bio‐let‐7g‐5p‐wt group than in the bio‐probe the NC group (*P* < .05). In addition, the HMGA2 mRNA expression did not differ significantly between the bio‐probe NC group and the bio‐let‐7g‐5p‐mut group (*P* > .05). These results indicated that bio‐let‐7g‐5p‐wt could promote the enrichment of HMGA2 mRNA around it while bio‐let‐7g‐5p‐mut could not.

### 3.5 VB and down‐regulation of HMGA2 suppress GBM cells migration and invasion

**Figure 5:**
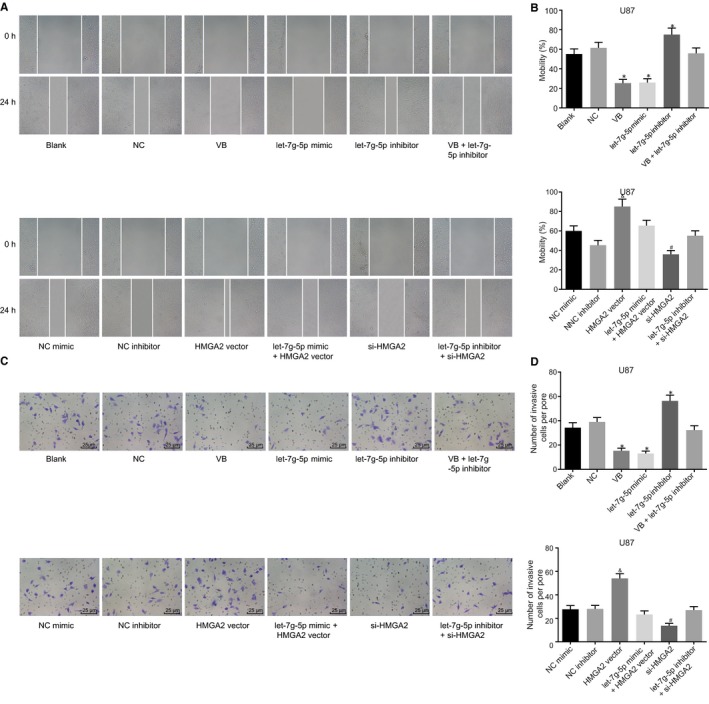
VB and HMGA2 blockade inhibit U87 cell migration and invasion in GBM. A, cell width at the 0th and 24th h in each group (×40); B, migration rate of U87 cells transfected with let‐7g‐5p mimic and/or HMGA2 vector, let‐7g‐5p inhibitor and/or si‐HMGA2 (n = 3, means ± SD); C, images of cell invasion in each group (×40); D, invasive U87 cells transfected with let‐7g‐5p mimic and/or HMGA2 vector, let‐7g‐5p inhibitor and/or si‐HMGA2 (n = 3, means ± SD); ^*^ *P* < .05 vs the blank group and the NC group; ^&^ *P* < .05 vs the NC mimic group; ^#^ *P* < .05 vs the NC inhibitor group. All measurement data were summarized as mean ± SD. Comparisons among multiple groups were performed with one‐way analysis of variance. The experiment was repeated three times independently

In the following experiments, we mainly investigated the effects of VB on the migration and invasion of GBM cells by using a scratch test model. The results of the U87 (Figure 5A,B) cells showed that in comparison with the blank and the NC groups, cell migration was decreased in the VB group and the let‐7g‐5p mimic group, increased in the let‐7g‐5p inhibitor group (all *P* < .05), but did not differ significantly in the VB + let‐7g‐5p inhibitor group (*P* > .05). No difference of cell migration was detected between the NC mimic group and NC inhibitor group (*P* > .05). In comparison with the NC mimic group, cell migration in the HMGA2 vector group exhibited elevated levels (*P* < .05). However, it did not differ significantly in the let‐7g‐5p mimic + the HMGA2 vector group (*P* > .05). In contrast to the NC inhibitor group, cell migration was decreased in the si‐HMGA2 group (*P* < .05), while it did not differ in the let‐7g‐5p inhibitor + si‐HMGA2 group (*P* > .05). The results of U251 cell experiments showed trends that were consistent with those observed in the U87 cells (Figure S1A,B).

The results of the transwell assay results using the U87 cells (Figure 5C,D) displayed a consistent trend. In comparison with the blank group and the NC group, cell invasion was decreased in the VB group and the let‐7g‐5p mimic group but increased in the let‐7g‐5p inhibitor group (all *P* < .05), while it did not differ significantly in the VB + let‐7g‐5p inhibitor group (*P* > .05). No difference of cell invasion was found between the NC mimic group and the NC inhibitor group (*P* > .05). In comparison with the NC mimic group, cell invasion in the HMGA2 vector group exhibited elevated levels (*P* < .05). However, cell invasion did not differ in the let‐7g‐5p mimic + HMGA2 vector group (*P* > .05). In contrast to the NC inhibitor group, cell invasion was decreased in the si‐HMGA2 group (*P* < .05), while no difference was observed in the let‐7g‐5p inhibitor + si‐HMGA2 group (*P* > .05). The results of the U251 cell experiments were similarly consistent with those of U87 cells (Figure S1C,D).

These data suggested that VB application, up‐regulation of let‐7g‐5p, as well as down‐regulation of HMGA2 each served to inhibit the cell migration and invasion in GBM cells.

### 3.6 VB promotes autophagy of U87 and U251 cells through the suppression of HMGA2 and the Wnt/β‐catenin signalling pathway

To further elucidate the role of VB and HMGA2 in relation to protein expression and autophagy of U87 and U251 cell lines, the expression of let‐7g‐5p, Beclin1, β‐catenin and HMGA2 in GBM cells was determined by laser confocal microscopy, RT‐qPCR and Western blot analysis.

**Figure 6:**
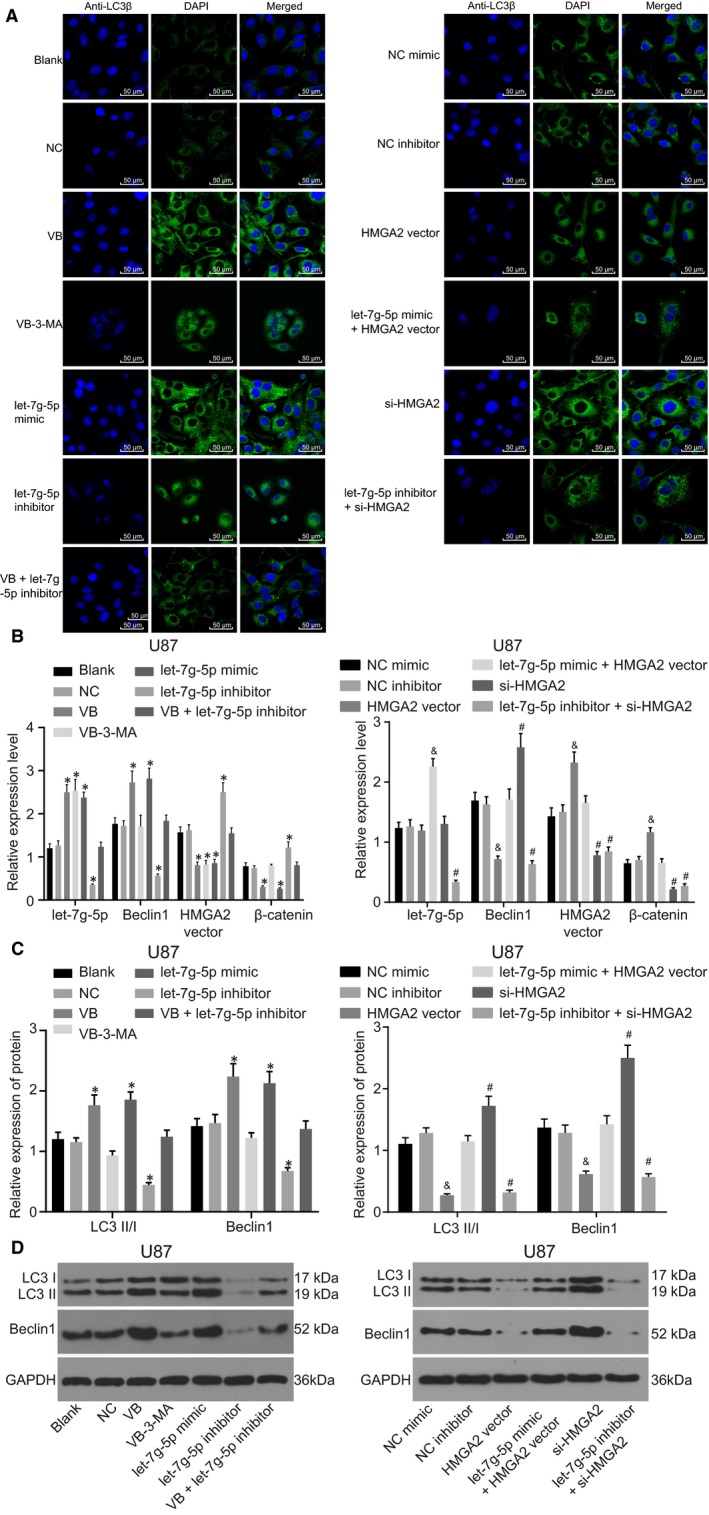
VB inhibits the Wnt/β‐catenin signalling pathway and promotes autophagy of U87 cell through suppression of HMGA2. A, the number of autophagosomes in U87 cells transfected with let‐7g‐5p mimic and/or HMGA2 vector, let‐7g‐5p inhibitor and/or si‐HMGA2; B, let‐7g‐5p expression and the mRNA expression of Beclin1, HMGA2 and β‐catenin in U87 cells transfected with let‐7g‐5p mimic and/or HMGA2 vector, let‐7g‐5p inhibitor and/or si‐HMGA2; C, the protein expression of LC3II/I and Beclin1 in U87 cells transfected with let‐7g‐5p mimic and/or HMGA2 vector, let‐7g‐5p inhibitor and/or si‐HMGA2; D, the grey value of LC3 I, LC3 II and Beclin1 protein bands in U87 cells transfected with let‐7g‐5p mimic and/or HMGA2 vector, let‐7g‐5p inhibitor and/or si‐HMGA2; ^*^ *P* < .05 vs the blank group and the NC group; ^&^ *P* < .05 vs the NC mimic group; ^#^ *P* < .05 vs the NC inhibitor group. All measurement data were summarized as mean ± SD. Comparisons among multiple groups were performed with one‐way analysis of variance. The experiment was repeated three times independently

The laser confocal microscopy results revealed same trends for U87 and U251 cell lines (Figures 6A,B, S2A,B). In comparison with the blank and the NC groups, the number of autophagosomes was increased in the VB and the let‐7g‐5p mimic groups but decreased in the let‐7g‐5p inhibitor group (all *P* < .05), whereas the VB + let‐7g‐5p inhibitor group did not differ significantly (*P* > .05). The number of autophagosomes observed in the NC mimic and the let‐7g‐5p NC inhibitor groups showed no significant difference (*P* > .05). Compared with the NC mimic group, fewer autophagosomes were found in the HMGA2 vector group (*P* < .05) but no significant differences were observed in the let‐7g‐5p mimic + HMGA2 vector group (*P* > .05). In contrast to the NC inhibitor group, the si‐HMGA2 group showed increased numbers of autophagosomes (*P* < .05), but no significant difference was recorded in the let‐7g‐5p inhibitor + si‐HMGA2 group (*P* > .05).

RT‐qPCR demonstrated that U87 and U251 cells shared the same trend (Figures 6C, S2C). The expression of let‐7g‐5p and Beclin1 was much higher, while HMGA2 and β‐catenin were expressed at lower levels in the VB, VB‐3‐MA and let‐7g‐5p mimic groups in comparison with the blank and the NC groups (all *P* < .05). In comparison with the blank and the NC groups, the let‐7g‐5p inhibitor group exhibited significantly lower let‐7g‐5p and Beclin1 expression but higher HMGA2 and β‐catenin expression (all *P* < .05), whereas these expression levels in the VB + let‐7g‐5p inhibitor group did not differ significantly (*P* > .05). The expression of let‐7g‐5p, Beclin1, HMGA2 and β‐catenin showed no difference in the NC mimic group and the NC inhibitor group (all *P* > .05). In comparison with the NC mimic group, the HMGA2 vector group showed repressed expression of Beclin1, while that of HMGA2 and β‐catenin was found elevated (all *P* < .05), with no differences in the expression of let‐7g‐5p (*P* > .05). In the let‐7g‐5p mimic + HMGA2 vector group, let‐7g‐5p expression was higher (*P* < .05), while no difference was observed in expression of HMGA2, Beclin1 and β‐catenin (all *P* > .05). In the si‐HMGA2 group, as compared to the NC inhibitor group, Beclin1 expression was enhanced, while suppression of HMGA2 and β‐catenin expression was observed (*P* < .05), and while let‐7g‐5p expression showed no difference (*P* > .05). The let‐7g‐5p inhibitor + si‐HMGA2 group displayed decreased let‐7g‐5p, Beclin1, β‐catenin and HMGA2 expression (all *P* < .05). Together these results indicated that VB enhanced let‐7g‐5p expression while it inhibited HMGA2 expression and the Wnt/β‐catenin signalling pathway. Additionally, let‐7g‐5p was found as capable of negatively regulating HMGA2, while overexpression of HMGA2 could further lead to activation of Wnt/β‐catenin signalling.

Western blot analysis revealed that U87 and U251 cells shared the same trend (Figures 6C,D, S2C,D). In comparison with the blank group and the NC group, LC3II/I and Beclin1 expression was much higher in the VB and the let‐7g‐5p mimic groups (*P* < .05), lower in the VB‐3‐MA and let‐7g‐5p inhibitor groups (*P* < .05), while no difference was observed in the VB + let‐7g‐5p inhibitor group (*P* > .05). The expression of LC3II/I and Beclin1 did not differ significantly in the NC mimic group and the NC inhibitor group (all *P* > .05). In contrast to the NC mimic group, LC3II/I and Beclin1 expression was lower in the HMGA2 vector group (*P* < .05) but showed no difference in the let‐7g‐5p mimic + HMGA2 vector group (*P* > .05). In comparison with the NC inhibitor group, the si‐HMGA2 group showed much higher LC3II/I and Beclin1 expression levels (*P* < .05) but these were significantly lower in the let‐7g‐5p inhibitor + si‐HMGA2 group (*P* < .05). These results suggested that let‐7g‐5p facilitated autophagy while overexpression of HMGA2 repressed autophagy.

### 3.7 VB promotes apoptosis of GBM cells

**Figure 7:**
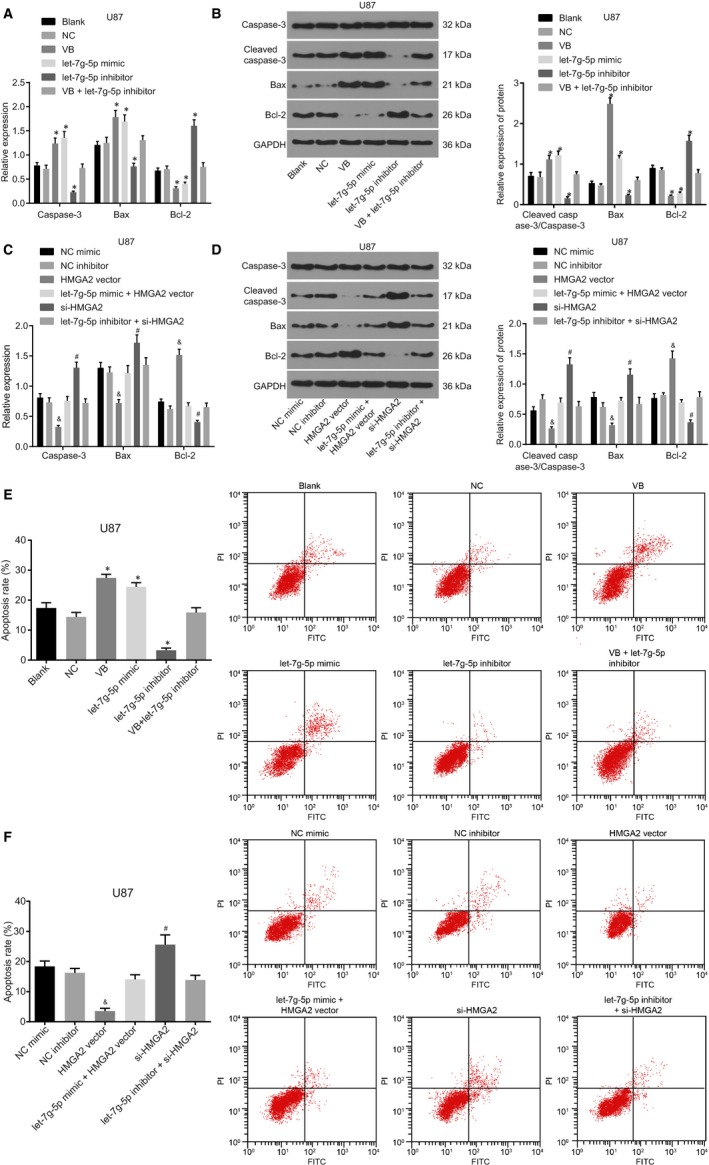
VB promotes cell apoptosis of U87 cells. A, mRNA expression of Caspase‐3, Bax and Bcl‐2; B, the protein expression of Caspase‐3, Bax and Bcl‐2, and the ratio of Cleaved caspase‐3/Caspase‐3 in U87 cells after transfection; C, the mRNA expression of Caspase‐3, Bax and Bcl‐2; D, the protein expression of Caspase‐3, Bax and Bcl‐2, and the ratio of Cleaved caspase‐3/Caspase‐3 in U87 cells after transfection; E, apoptosis rate of U87 cells transfected with let‐7g‐5p mimic, let‐7g‐5p inhibitor alone or in the presence of VB; F, apoptosis rate of U87 cells transfected with HMGA2 vector alone or in the presence of let‐7g‐5p mimic, si‐HMGA2 alone or in the presence of let‐7g‐5p inhibitor; ^*^ *P* < .05 vs the blank group and the NC group; ^&^ *P* < .05 vs the NC mimic group; ^#^ *P* < .05 vs the NC inhibitor group. All measurement data were summarized as mean ± SD. Comparisons among multiple groups were performed with one‐way analysis of variance. The experiment was repeated three times independently

An investigation into the effect of VB on cell apoptosis was conducted using RT‐qPCR, Western blot analysis and flow cytometry. RT‐qPCR (Figure 7A and C) revealed that expression of Bax and Caspase‐3 was much higher, while the expression of Bcl‐2 was lower in the VB and the let‐7g‐5p mimic groups, in comparison with the blank and the NC group (all *P* < .05). In comparison with the blank and NC groups, the let‐7g‐5p inhibitor group exhibited significantly lower expression of Bax and Caspase‐3, with higher expression of Bcl‐2 (all *P* < .05). Expression of genes in the VB + let‐7g‐5p inhibitor group did not differ significantly from the blank and NC groups (*P* > .05). The expression of Bax, Caspase‐3 and Bcl‐2 showed no difference between the NC mimic group and the NC inhibitor group (*P* > .05). In comparison with the NC mimic group, in the HMGA2 vector group, the expression of Bax and Caspase‐3 was repressed, and Bcl‐2 mRNA expression was increased (all *P* < .05). Additionally, no differences were recorded in the expression of Bax, Caspase‐3 and Bcl‐2 in the let‐7g‐5p mimic + HMGA2 vector group (all *P* > .05). In the si‐HMGA2 group, in contrast to the NC inhibitor group, the expression of Bax and Caspase‐3 was enhanced, with suppressed expression of Bcl‐2 (all *P* < .05) and no significant difference in expression of let‐7g‐5p (*P* > .05). In the let‐7g‐5p inhibitor + si‐HMGA2 group, expression of Bax, Caspase‐3 and Bcl‐2 did not differ significantly (all *P* > .05). Similar trends were noted in both U87 and U251 cell experiments (Figure S3A and C).

Western blot analysis results revealed that the U87 and U251 cells shared the same trend (Figures 7B and D, S3 B and D). In contrast to the blank group and the NC group, expression of Bax and Cleaved caspase‐3/Caspase‐3 was much higher, while that of Bcl‐2 was lower in the VB and the let‐7g‐5p mimic group (all *P* < .05). In the let‐7g‐5p inhibitor group, expression of Bax and Cleaved caspase‐3/Caspase‐3 was much lower, while that of Bcl‐2 was higher (all *P* < .05). The expression of Bax, Cleaved caspase‐3/Caspase‐3 and Bcl‐2 showed no differences in the VB + let‐7g‐5p inhibitor group (*P* > .05) and did not differ significantly between the NC mimic and the NC inhibitor groups (*P* > .05). In the HMGA2 vector group, as compared to the NC mimic group, expression of Bax and Cleaved caspase‐3/Caspase‐3 was diminished, while Bcl‐2 expression was enhanced (all *P* < .05). No significant difference was found in the let‐7g‐5p mimic + HMGA2 vector group (all *P* > .05). In the si‐HMGA2 group, in comparison with the NC inhibitor group, Bax and Cleaved caspase‐3/Caspase‐3 expression was elevated, while Bcl‐2 was reduced (all *P* < .05). No differences in expression of Bax, Cleaved caspase‐3/Caspase‐3 and Bcl‐2 were exhibited in the let‐7g‐5p inhibitor + si‐HMGA2 group (all *P* > .05).

Flow cytometry results suggested parallel trends in U87 and U251 cells (Figures 7E‐F, S3E‐F). In comparison with the blank and NC groups, cell apoptosis was increased in the VB and let‐7g‐5p mimic groups, but decreased in the let‐7g‐5p inhibitor group (all *P* < .05). In addition, the VB + let‐7g‐5p inhibitor group did not differ significantly (*P* > .05). No significant difference was observed in cell apoptosis between the NC mimic and the inhibitor groups (*P* > .05). In the HMGA2 vector group, as compared with the NC mimic group, cell apoptosis was decreased (*P* < .05). No difference in the let‐7g‐5p mimic + HMGA2 vector group was noted (*P* > .05). In the si‐HMGA2 group, as compared to the NC inhibitor group, cell apoptosis was increased (*P* < .05), whereas the let‐7g‐5p inhibitor + si‐HMGA2 group did not display any significant difference (*P* > .05). Overall, these results indicated that overexpression of let‐7g‐5p or knockdown of HMGA2 potentiated the apoptosis of GBM cells.

### 3.8 VB impairs tumour growth in GBM through up‐regulation of let‐7g‐5p

**Figure 8:**
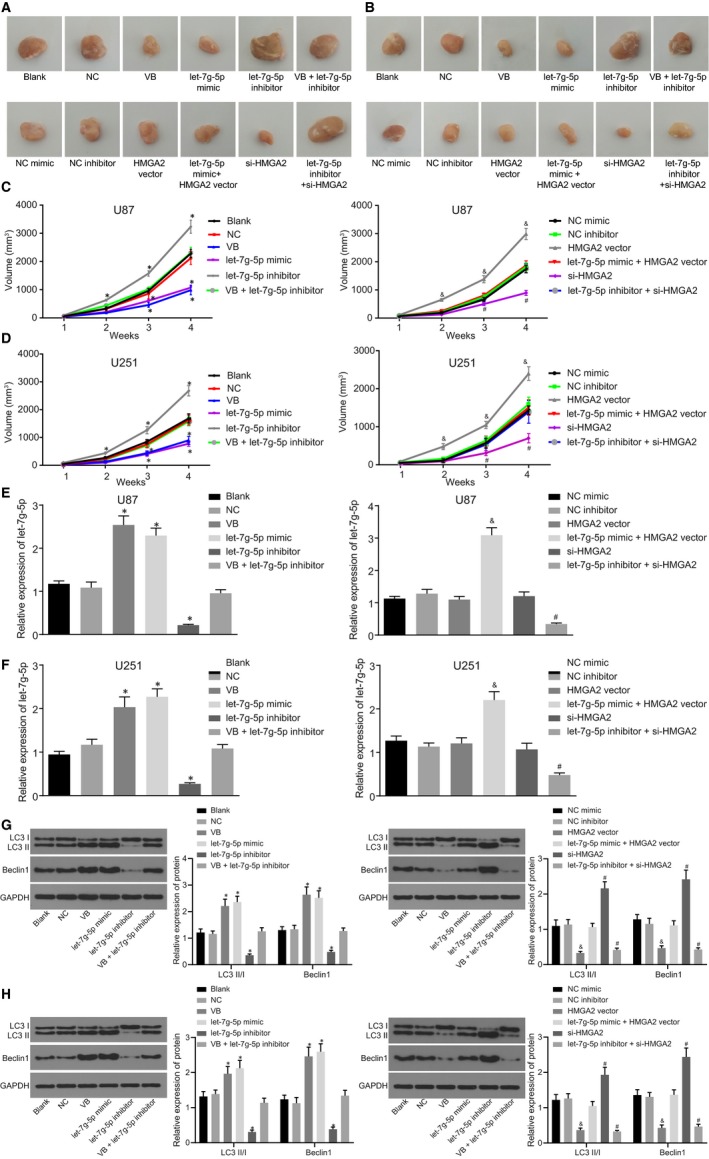
VB represses tumour growth of GBM cells by up‐regulation of let‐7g‐5p in vivo. A, xenograft tumours in nude mice injected with U87 cells in each group; B, xenograft tumours in nude mice injected with U251 cells in each group; C, changes of tumour volume in nude mice injected with U87 cells at 1, 2, 3, and 4 w; D, changes of tumour volume in nude mice injected with U251 cells at 1, 2, 3 and 4 w; E, let‐7g‐5p expression in nude mice injected with U87 cells in each group by RT‐qPCR; F, let‐7g‐5p expression in nude mice injected with U251 cells in each group by RT‐qPCR; G, expression of LC3 II/I and Beclin1 after U87 tumorigenesis in each group measured by Western blot; H, expression of LC3 II/I and Beclin1 after U251 tumorigenesis in each group measured by Western blot.^*^ *P* < .05 vs the blank group and the NC group; ^&^ *P* < .05 vs the NC mimic group; ^#^ *P* < .05 vs the NC inhibitor group. All measurement data were summarized as mean ± SD. Comparisons among multiple groups were performed with one‐way analysis of variance. The experiment was repeated three times independently

Next, GBM xenograft tumours established in nude mice were used to determine the effect of VB on the tumour growth of GBM in vivo and revealed similar results for U87 and U251 cells (Figure 8A‐D). In comparison with the blank and the NC groups, tumour growth was inhibited in the VB and let‐7g‐5p mimic groups, but was promoted in the let‐7g‐5p inhibitor group (all *P* < .05). No significant difference was detected in the VB + let‐7g‐5p inhibitor group (*P* > .05). These results demonstrated the ability of VB to suppress the growth of GBM tumours. Tumour growth showed no difference between the NC mimic and the NC inhibitor groups (*P* > .05). In comparison with the NC mimic group, tumour growth was accelerated in the HMGA2 vector group (*P* < .05) but no difference was detected in the let‐7g‐5p mimic + HMGA2 vector group (*P* > .05). As compared with the NC inhibitor group, tumour growth was inhibited (*P* < .05) in the si‐HMGA2 group, whereas the let‐7g‐5p inhibitor + si‐HMGA2 group did not display any notable difference (*P* > .05).

The RT‐qPCR results also indicated that U87 and U251 cells showed similar trends (Figure 8E‐F). In comparison with the blank and the NC groups, the expression of let‐7g‐5p was elevated in the VB and the let‐7g‐5p mimic groups, but significantly reduced in the let‐7g‐5p inhibitor group (all *P* < .05). However, no distinct difference was detected in the VB + let‐7g‐5p inhibitor group (*P* > .05). These results also indicated that VB promoted the expression of let‐7g‐5p and inhibited tumour growth of GBM cells. No difference was observed in tumour volume in the NC mimic and the NC inhibitor groups (*P* > .05). In comparison with the NC mimic group, let‐7g‐5p expression did not significantly differ in the HMGA2 vector group (*P* > .05). However, let‐7g‐5p was significantly elevated in the let‐7g‐5p mimic + HMGA2 vector group (*P* < .05). In contrast to the NC inhibitor group, let‐7g‐5p expression did significantly differ in the si‐HMGA2 vector group (*P* > .05), but was significantly inhibited in the let‐7g‐5p inhibitor + si‐HMGA2 group (*P* < .05).

Expression of LC3II/I and Beclin1 in tumours was further examined by Western blot. The results showed that as compared with the blank and NC groups, LC3II/I and Beclin1 were increased in the VB and let‐7g‐5p mic groups, but reduced in the let‐7g‐5p inhibitor group (*P* < .05), while there was no significant difference in the VB + let‐7g‐5p inhibitor group (*P* > .05). In the HMGA2 vector group, the expression of LC3 II/I and Beclin1 was significantly decreased (*P* < .05), but was reversed in the let‐7g‐5p mimic + HMGA2 vector group. The si‐HMGA2 group showed significantly increased expression of LC3 II/I and Beclin1 (*P* < .05), while let‐7g‐5p inhibitor + si‐HMGA2 group showed significantly decreased expression (*P* < .05) (Figure 8G‐H).

The findings displayed that VB‐mediated let‐7g‐5p might inhibit the tumour growth of GBM in vivo via down‐regulating HMGA2.

### 3.9 VB blocks the PKC pathway through let‐7g‐5p

**Figure 9:**
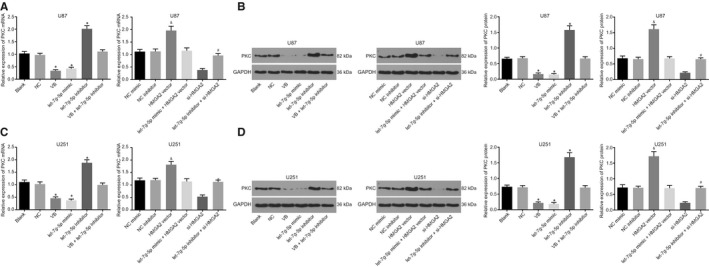
VB blocks the expression of PKC by up‐regulation of let‐7g‐5p in GBM cells. A, the mRNA expression of PKC in U87 cells after transfection; B, the protein expression of PKC in U87 cells after transfection; C, the mRNA expression of PKC in U251 cells after transfection; D, the protein expression of PKC in U251 cells after transfection; ^*^ *P* < .05 vs the blank group and the NC group; ^&^ *P* < .05 vs the NC mimic group or the NC inhibitor group; ^#^ *P* < .05 vs the HMGA2 vector group or the si‐HMGA2 group. All measurement data were summarized as mean ± SD. Comparisons among multiple groups were performed with one‐way analysis of variance. The experiment was repeated three times independently

As previous findings have indicated, VB promotes carcinogenesis by inhibiting the PKC pathway.^23^ PKC being a Wnt signalling pathway‐related gene, we endeavoured, to determine whether VB could affect Wnt signalling pathway by down‐regulating PKC. For this objective, we measured the mRNA and protein expression of PKC in GBM cells by RT‐qPCR and Western blot analysis, respectively. The results showed that there was no significant difference in the expression of PKC between the blank and NC groups (*P* > .05). As compared with the blank and NC groups, the VB and let‐7g‐5p mimic groups demonstrated decreased expression of PKC (*P* < .05), while the let‐7g‐5p inhibitor group showed significantly enhanced PKC expression (*P* < .05). As compared with VB treatment alone, the mRNA and protein expression of PKC increased after combined treatment with VB and let‐7g‐5p inhibitor. There was no significant difference in PKC expression between the NC mimic and NC inhibitor groups (*P* > .05), but the mRNA and protein expression of PKC significantly increased upon HMGA2 vector treatment (*P* < .05), which was reversed by the addition of let‐7g‐5p mimic (*P* > .05). The mRNA and protein expression of PKC in the si‐HMGA2 group was significantly diminished (*P* < .05), whereas PKC expression in the let‐7g‐5p inhibitor + si‐HMGA2 group was relatively higher than that in the si‐HMGA2 group (Figure 9A,B). The results of U87 and U251 cell experiments were consistent (Figure 9C,D). These findings showed PKC could be blocked by VB‐mediated let‐7g‐5p overexpression in GBM cells.

## 4 DISCUSSION

GBM is a prototypical heterogeneous brain tumour that exhibits significant resistance to routine treatment and is frequently marked by a devastating rate of tumour recurrence.^24^ Previous studies have shown that VB has protective effects against colorectal cancer and leukaemia, where it modulates the biological processes involved.^9^, ^25^ Yet the effect of VB remains poorly understood. In the present study, the main objectives were to identify the let‐7g‐5p, HMGA2 axis, acting via the Wnt/β‐catenin pathway, as a novel regulatory mode of action of VB, as well as characterize the effects of VB on GBM cells. Altogether, our study demonstrated that VB promotes let‐7g‐5p expression, which down‐regulates HMGA2 via Wnt/β‐catenin signalling blockade, and results in inhibited GBM tumour cell proliferation and promoted autophagy.

A key observation of this study is the ability of VB to enhance let‐7g‐5p expression and inhibit HMGA2 as well as Wnt/β‐catenin signalling. Our experimental data initially demonstrated that VB could up‐regulate let‐7g‐5p in GBM cell lines. VB is considered to have significant clinical value from a cancer perspective, owing to its neuroprotective and memory enhancement properties.^26^ In agreement with our data, Areeb *et al* illustrated that let‐7g‐5p was significantly poorly expressed in patients with GBM.^27^ The in silico target prediction and the luciferase activity assay demonstrated HMGA2 was a putative target gene of let‐7g‐5p, which was negatively governed by let‐7g‐5p. Studies have reported that the miR‐let‐7 family is associated with HMGA2, through which it co‐regulates many cancers. For instance, let‐7 was found to act as a tumour suppressor by targeting HMGA2 in colorectal cancer, in a report by Madison et al.^28^ Other experiments indicated that H19 promoted pancreatic ductal adenocarcinoma cell invasion and migration by activating let‐7‐mediated HMGA2.^29^ In the present study, we demonstrated that HMGA2 activated the Wnt/β‐catenin signalling pathway in GBM cells. Consistent with our findings, previous work in gastric cancer has shown that HMGA2 enhances epithelial‐mesenchymal transitions via the activation of the Wnt/β‐catenin signalling pathway.^30^ Similarly, in hepatocellular carcinoma, the Wnt/β‐catenin signalling pathway has been found to be activated by HMGA2, which results in inhibited tumour cell proliferation.^31^

The most significant finding of our study is that VB treatment or up‐regulation of let‐7g‐5p could repress GBM tumour cell viability, invasion and migration, while enhancing cell apoptosis and autophagy. In contrast, the overexpression of HMGA2 or activation of the Wnt/β‐catenin signalling pathway led to the opposite results. Consistent with our findings, VB was shown to suppress cell proliferation in gastric cancer.^32^ Hugo et al demonstrated that VB was protective against prostate cancer, acting to reduce the levels of reactive oxygen species in cancer cells.^33^ Zhou et al similarly found that VB up‐regulated colorectal cancer cell apoptosis by activating the HIPK2‐p53 signalling pathway.^25^ Notably, our in vitro experiments indicated that VB could down‐regulate PKC, a Wnt pathway‐related gene, by modulating the expression of let‐7g‐5p. Its aberrant expression in GBM is noted to regulate cancer cell proliferation, apoptosis and invasiveness through downstream influence on multiple genes.^34^ Zhang et al further elucidated that the up‐regulation of let‐7g‐5p inhibits cell invasion and migration in GBM U‐87MG cells, acting via a reduction in VSIG4.^10^ HMGA2 is generally considered as a tumour promoter gene in several cancers. For instance, HMGA2 enhances lung carcinogenesis, serving as a protein‐coding gene.^12^ In a similar finding, Zhao et al noted HMGA2 overexpression in tongue cancer was associated with metastasis.^35^ In GBM particularly, recent evidence has demonstrated that HMGA2 accelerates both tumour cell line stemness and cell invasion.^36^ Abnormal Wnt/β‐catenin signalling is shown to promote GBM carcinogenesis and progression.^37^, ^38^ In accordance, the inhibition of Wnt/β‐catenin signalling has been reported to negatively impact cell proliferation, migration and invasion in GBM.^17^ Others have reported the activation of the Wnt/β‐catenin signalling accelerates stemness of GBM cells.^39^

To conclude, the current study provides evidence demonstrating that VB suppresses GBM cell viability, invasion and migration, while enhancing cell apoptosis and autophagy. These effects are achieved through the inhibition of let‐7g‐5p‐mediated HMGA2 and blockade of the Wnt/β‐catenin signalling pathway. These results suggest that VB may be a promising treatment modality for GBM. In addition, our findings also suggest that let‐7g‐5p, HMGA2 and the Wnt/β‐catenin signalling pathway‐related genes could have potential as predictive biomarkers for GBM. However, further experiments are needed to unravel the detailed mechanisms by which VB may cross the blood‐brain barrier. In addition, investigations using orthotopic models are warranted in order to further validate the findings of the present study.

## CONFLICT OF INTEREST

The authors confirm that there are no conflicts of interest.

## AUTHORS’ CONTRIBUTIONS

Wei‐Qiang Jia, Cheng‐Yong Yang and Jun Ma conceived and designed the experiments. Tian‐You Pu participated in its design and coordination. Jun Ma and Ming‐Ming Zou performed the experiments and the statistical analysis. Wei‐Qiang Jia, Cheng‐Yong Yang, Guo‐Qiang Han drafted the manuscript. Jian‐Wei Zhu and Ru‐Xiang Xu edited and revised the manuscript. All authors read and approved the final manuscript.

## Data Availability

The data that support the findings of this study are available from the corresponding author upon reasonable request.

## References

[1] Komotar RJ , Otten ML , Moise G , et al. Radiotherapy plus concomitant and adjuvant temozolomide for glioblastoma‐a critical review. Clin Med Oncol. 2008;2:421‐422. 21892310 10.4137/cmo.s390 PMC3161656

[2] Stupp R , Taillibert S , Kanner AA , et al. Maintenance therapy with tumor‐treating fields plus temozolomide vs temozolomide alone for glioblastoma: a randomized clinical trial. JAMA. 2015;314:2535‐2543. 26670971 10.1001/jama.2015.16669

[3] Joseph JV , Conroy S , Pavlov K , et al. Hypoxia enhances migration and invasion in glioblastoma by promoting a mesenchymal shift mediated by the HIF1alpha‐ZEB1 axis. Cancer Lett. 2015;359:107‐116. 25592037 10.1016/j.canlet.2015.01.010

[4] Yao Y , Ma J , Xue Y , et al. Knockdown of long non‐coding RNA XIST exerts tumor‐suppressive functions in human glioblastoma stem cells by up‐regulating miR‐152. Cancer Lett. 2015;359:75‐86. 25578780 10.1016/j.canlet.2014.12.051

[5] Carlsson SK , Brothers SP , Wahlestedt C . Emerging treatment strategies for glioblastoma multiforme. EMBO Mol Med. 2014;6:1359‐1370. 25312641 10.15252/emmm.201302627 PMC4237465

[6] Alipieva KKL , Orhan IE , Georgiev MI . Verbascoside–a review of its occurrence, (bio)synthesis and pharmacological significance. Biotechnol Adv. 2014;32:1065‐1076. 25048704 10.1016/j.biotechadv.2014.07.001

[7] Isacchi B , Bergonzi MC , Iacopi R , et al. Liposomal formulation to increase stability and prolong antineuropathic activity of verbascoside. Planta Med. 2017;83:412‐419. 27191581 10.1055/s-0042-106650

[8] Zhu M , Tan N , Zhu H , et al. Anti‐sports anaemia effects of verbascoside and martynoside in mice. Int J Sports Med. 2010;31:537‐541. 20556696 10.1055/s-0030-1254115

[9] Zhang Y , Liu B , Wu H , et al. Anti‐tumor activity of verbascoside loaded gold nanoparticles. J Biomed Nanotechnol. 2014;10:3638‐3646. 26000377 10.1166/jbn.2014.2052

[10] Zhang XH , Qian Y , Li Z , et al. Let‐7g‐5p inhibits epithelial‐mesenchymal transition consistent with reduction of glioma stem cell phenotypes by targeting VSIG4 in glioblastoma. Oncol Rep. 2016;36:2967‐2975. 27634309 10.3892/or.2016.5098

[11] Liu Q , Lv GD , Qin X , et al. Role of microRNA let‐7 and effect to HMGA2 in esophageal squamous cell carcinoma. Mol Biol Rep. 2012;39:1239‐1246. 21598109 10.1007/s11033-011-0854-7

[12] Kumar MSA‐ME , East P , Chakravorty P , Matthews N , Winslow MM . HMGA2 functions as a competing endogenous RNA to promote lung cancer progression. Nature. 2014;505:212‐217. 24305048 10.1038/nature12785 PMC3886898

[13] Morishita AZM , Mitoro A , Sankarasharma D , Szabolcs M , Okada Y . HMGA2 is a driver of tumor metastasis. Cancer Res. 2013;73:4289‐4299. 23722545 10.1158/0008-5472.CAN-12-3848 PMC3715567

[14] Zhu C , Li J , Cheng G , et al. miR‐154 inhibits EMT by targeting HMGA2 in prostate cancer cells. Mol Cell Biochem. 2013;379:69‐75. 23591597 10.1007/s11010-013-1628-4

[15] Wang YCF , Zhao M , Yang Z , Zhang S , Ye L . MiR‐107 suppresses proliferation of hepatoma cells through targeting HMGA2 mRNA 3'UTR. Biochem Biophys Res Commun. 2016;480:455‐460. 27773820 10.1016/j.bbrc.2016.10.070

[16] Kim KHSH , Kim EH , Rheey J , Jin HJ , Lee Y . Wnt/beta‐catenin signaling is a key downstream mediator of MET signaling in glioblastoma stem cells. Neuro Oncol. 2013;15:161‐171. 23258844 10.1093/neuonc/nos299 PMC3548587

[17] Liu X , Gao Q , Zhao N , et al. Sohlh1 suppresses glioblastoma cell proliferation, migration, and invasion by inhibition of Wnt/beta‐catenin signaling. Mol Carcinog. 2018;57:494‐502. 29240260 10.1002/mc.22774

[18] Ayuk SM , Abrahamse H , Houreld NN . The role of photobiomodulation on gene expression of cell adhesion molecules in diabetic wounded fibroblasts in vitro. J Photochem Photobiol B. 2016;161:368‐374. 27295416 10.1016/j.jphotobiol.2016.05.027

[19] Li J , Pandey V , Kessler T , et al. Modeling of miRNA and drug action in the EGFR signaling pathway. PLoS ONE. 2012;7:e30140. 22253908 10.1371/journal.pone.0030140 PMC3256223

[20] Wu L , Pu X , Wang Q , et al. miR‐96 induces cisplatin chemoresistance in non‐small cell lung cancer cells by downregulating SAMD9. Oncol Lett. 2016;11:945‐952. 26893673 10.3892/ol.2015.4000 PMC4734049

[21] Wu K , Yang Y , Zhao J , et al. BAG3‐mediated miRNA let‐7g and let‐7i inhibit proliferation and enhance apoptosis of human esophageal carcinoma cells by targeting the drug transporter ABCC10. Cancer Lett. 2016;371:125‐133. 26655271 10.1016/j.canlet.2015.11.031

[22] Petrillo M , Zannoni GF , Beltrame L , et al. Identification of high‐grade serous ovarian cancer miRNA species associated with survival and drug response in patients receiving neoadjuvant chemotherapy: a retrospective longitudinal analysis using matched tumor biopsies. Ann Oncol. 2016;27:625‐634. 26782955 10.1093/annonc/mdw007

[23] Herbert JM , Maffrand JP , Taoubi K , et al. Verbascoside isolated from Lantana camara, an inhibitor of protein kinase C. J Nat Prod. 1991;54:1595‐1600. 1812212 10.1021/np50078a016

[24] Kim H , Zheng S , Amini SS , et al. Whole‐genome and multisector exome sequencing of primary and post‐treatment glioblastoma reveals patterns of tumor evolution. Genome Res. 2015;25:316‐327. 25650244 10.1101/gr.180612.114 PMC4352879

[25] Zhou L , Feng Y , Jin Y , et al. Verbascoside promotes apoptosis by regulating HIPK2‐p53 signaling in human colorectal cancer. BMC Cancer. 2014;14:747. 25282590 10.1186/1471-2407-14-747 PMC4197337

[26] Gao L , Peng XM , Huo SX , et al. Memory enhancement of acteoside (Verbascoside) in a senescent mice model induced by a combination of D‐gal and AlCl3. Phytother Res. 2015;29:1131‐1136. 25900014 10.1002/ptr.5357

[27] Areeb ZSS , Koldej R , Ritchie DS , Siegal T , Morokoff AP . MicroRNA as potential biomarkers in Glioblastoma. J Neurooncol. 2015;125:237‐248. 26391593 10.1007/s11060-015-1912-0

[28] Madison BB , Jeganathan AN , Mizuno R , et al. Let‐7 Represses carcinogenesis and a stem cell phenotype in the intestine via regulation of Hmga2. PLoS Genet. 2015;11:e1005408. 26244988 10.1371/journal.pgen.1005408 PMC4526516

[29] Ma C , Nong K , Zhu H , et al. H19 promotes pancreatic cancer metastasis by derepressing let‐7's suppression on its target HMGA2‐mediated EMT. Tumour Biol. 2014;35:9163‐9169. 24920070 10.1007/s13277-014-2185-5

[30] Zha L , Zhang J , Tang W , et al. HMGA2 elicits EMT by activating the Wnt/beta‐catenin pathway in gastric cancer. Dig Dis Sci. 2013;58:724‐733. 23135750 10.1007/s10620-012-2399-6

[31] Ou W , Lv J , Zou X , et al. Propofol inhibits hepatocellular carcinoma growth and invasion through the HMGA2‐mediated Wnt/beta‐catenin pathway. Exp Ther Med. 2017;13:2501‐2506. 28565871 10.3892/etm.2017.4253 PMC5443290

[32] Hsu HF , Houng JY , Kuo CF , et al. Glossogin, a novel phenylpropanoid from Glossogyne tenuifolia, induced apoptosis in A549 lung cancer cells. Food Chem Toxicol. 2008;46:3785‐3791. 18976690 10.1016/j.fct.2008.09.068

[33] Garro HAGC , Martin VS , Tonn CE , Pungitore CR . A new iridoid, verbascoside and derivatives with inhibitory activity against Taq DNA polymerase. Bioorg Med Chem Lett. 2015;25:914‐918. 25582597 10.1016/j.bmcl.2014.12.052

[34] Shi L , Chen J , Yang J , et al. MiR‐21 protected human glioblastoma U87MG cells from chemotherapeutic drug temozolomide induced apoptosis by decreasing Bax/Bcl‐2 ratio and caspase‐3 activity. Brain Res. 2010;1352:255‐264. 20633539 10.1016/j.brainres.2010.07.009

[35] Zhao XPZH , Jiao JY , Tang DX , Wu YL , Pan CB . Overexpression of HMGA2 promotes tongue cancer metastasis through EMT pathway. J Transl Med. 2016;14:26. 26818837 10.1186/s12967-016-0777-0 PMC4730598

[36] Kaur H , Ali SZ , Huey L , et al. The transcriptional modulator HMGA2 promotes stemness and tumorigenicity in glioblastoma. Cancer Lett. 2016;377:55‐64. 27102002 10.1016/j.canlet.2016.04.020 PMC5091648

[37] Huang JXD , Li G , Ma J , Chen P , Yuan W . EphA2 promotes epithelial‐mesenchymal transition through the Wnt/beta‐catenin pathway in gastric cancer cells. Oncogene. 2014;33:2737‐2747. 23752181 10.1038/onc.2013.238

[38] Ma FZJ , Zhong L , Wang L , Liu Y , Wang Y . Upregulated microRNA‐301a in breast cancer promotes tumor metastasis by targeting PTEN and activating Wnt/beta‐catenin signaling. Gene. 2014;535:191‐197. 24315818 10.1016/j.gene.2013.11.035

[39] Adamo AFD , De Martino F , Roscigno G , Affinito A , Donnarumma E . RYK promotes the stemness of glioblastoma cells via the WNT/ beta‐catenin pathway. Oncotarget. 2017;8:13476‐13487. 28086236 10.18632/oncotarget.14564 PMC5355113

